# Supplementary material for: Individual retrotransposon integrants are differentially controlled by KZFP/KAP1-dependent histone methylation, DNA methylation and TET-mediated hydroxymethylation in naïve embryonic stem cells
Source: Epigenetics Chromatin. 2018 Feb 26;11:7. doi: 10.1186/s13072-018-0177-1 (PMC6389204; doi:10.1186/s13072-018-0177-1)
Supplement: Supplementary file 11 — Additional file 11. Pattern analysis. [file 13072_2018_177_MOESM11_ESM.zip › Patterns analysis/DataTables/extensions/KeyTable/examples/index.html]

KeyTable examples - KeyTable examples


# KeyTable example KeyTable examples

KeyTable provides enhanced accessibility and navigation options for DataTables enhanced tables, by
allowing Excel like cell navigation on any table. Events (focus, blur, action etc) can be assigned to
individual cells, columns, rows or all cells to allow advanced interaction options.. Key features
include:

- Easy to use spreadsheet like interaction
- Fully integrated with DataTables
- Wide range of supported events
- Works without DataTables if you just want a plain table

### Examples

- Basic initialisation
- Events
- Scrolling table
- Plain HTML table

Please refer to the DataTables documentation for full
information about its API properties and methods.  
Additionally, there are a wide range of extras and
plug-ins which extend the capabilities of
DataTables.

DataTables designed and created by SpryMedia Ltd © 2007-2014  
DataTables is licensed under the MIT license.
